# Retrospective analysis of prognosis and risk factors of patients with stroke by TOAST

**DOI:** 10.1097/MD.0000000000010412

**Published:** 2018-04-13

**Authors:** Weimin Wei, Suting Li, Fulan San, Shijun Zhang, Qingyu Shen, Jianjun Guo, Li Zhang

**Affiliations:** a Department of Neurology; b Department of Emergency, The Zengcheng People's Hospital (Boji-Affiliated Hospital of Sun Yat-sen University), Guangzhou; c The First Affiliated Hospital of Guangdong Pharmaceutical University, Guangdong, China.

**Keywords:** etiologic subtype, ischemic stroke, prognosis, TOAST

## Abstract

To determine differences in 90-day mortality and identify risk factors among different etiological classifications of ischemic stroke using the Trial of ORG 10172 in Acute Stroke Treatment (TOAST) classification.

Our retrospective analysis included 538 ischemic stroke patients. The cause of stroke was categorized according to the TOAST criteria, and 90-day mortality rates were obtained through the patient follow-up. Age, sex, previous medical history, and clinical features were used in the analysis of potential risk factors.

There were 38 deaths during the 90-day follow-up period. Patients in the undetermined cause subgroups experienced significantly higher mortality rate than those in subgroups with small artery occlusion and large artery atherosclerosis. Factors independently associated with 90-day mortality for patients with the large artery atherosclerosis stroke subtype were age (95% confidence interval [CI], 1.010–1.192, *P* = .028), history of hypertension (95% CI, 3.030–99.136, *P* = .001), high blood glucose (95% CI, 1.273–2.354, *P* < .001), high cholesterol (95% CI, 0.017–0.462, *P* = .004), high uric acid (95% CI, 2.360–64.389, *P* = .003), and National Institute of Health Stroke Scale(95% CI, 1.076–1.312, *P* = .001). Age (95% CI, 1.012–1.358, *P* = .034) and high cholesterol (95% CI, 0.011–0.496, *P* = .007) were independently associated with 90-day mortality for patients with the small artery occlusion subtype of stroke.

Our analysis identified that certain risk factors and 90-day mortality differ significantly among different stroke subtypes, as classified by the TOAST criteria. These risk factors must be considered carefully to provide the best clinical management of these patients and thus reduce mortality.

## 1 Introduction

Stroke has recently overtaken cancer as the most common cause of death in China^[1]^ and can seriously affect patients’ quality of life and place a great burden upon both patients’ families and society. Ischemic stroke constitutes 87% of all strokes,^[2]^ and there is an urgent unmet need to identify risk factors associated with ischemic stroke for use in reducing the incidence, recurrence, morbidity, and mortality of this condition.

Ischemic stroke is a series of neurological syndromes with different etiological, clinical, and prognostic features. The exact causes and mechanisms of ischemic stroke remain unclear. A classification system for stroke subtypes that accounts for these differences is crucial and may lead to different treatments and indicators for the application of different secondary prevention measures.^[3,4]^

The Trial of ORG 10172 in Acute Stroke Treatment (TOAST) classification is a widely used method for classifying ischemic stroke and involves the division of stroke into different subtypes according to neurological signs, cerebral imaging, and ancillary diagnostic test results.^[5]^ Because of its ease of use in the clinic, the TOAST classification has proven to be very reliable. Over recent years, researchers have undertaken a range of studies on the clinic features, recurrence, and prognosis of ischemic stroke based upon TOAST classification.^[6,7]^ However, the specific risk factors associated with different TOAST subtypes of ischemic stroke have yet to be identified. Knowledge of the risk factors for ischemic stroke would permit medical intervention in the early stages of disease, which would allow for the reduction of the incidence of ischemic stroke. Consequently, this study aimed to investigate potential risk factors for different subtypes of ischemic stroke, as classified by the TOAST criteria, and to analyze their relative roles in the prognosis of stroke to provide better clinical guidance for the prevention and treatment of stroke.

## 2 Methods

### 2.1 Participants

We included 538 patients who were diagnosed with their first stroke in our hospital between January 2006 and January 2016. Our trial was approved by the local research ethics committee, and all participants or their authorized surrogate decision maker provided informed written consent. The inclusion criteria included stroke diagnosed and confirmed by computed tomography (CT) or magnetic resonance imaging (MRI), or disappearance of symptoms and signs within 24 hours but with lesions identified by CT or MRI and diagnosed with ischemic stroke; age between 50 and 90 years; and hospital admission within 7 days of the appearance of symptoms or clinical signs. Patients were excluded based on a prior diagnosis of primary intracerebral hemorrhage, intraventricular hemorrhage, or subarachnoid hemorrhage; inability to complete our coronal vascular imaging examination; severe cognitive impairment restricting participation in this study; and severe cardiopulmonary insufficiency, liver diseases, kidney diseases, and malignant tumors.

### 2.2 Trial of ORG 10172 in Acute Stroke Treatment

Ischemic stroke cases were categorized by subtype according to the original TOAST criteria. Within this classification, there are 5 major categories: large artery atherosclerosis (LAA), cardioembolism (CE), small artery occlusion (SAO), stroke of other determined cause (SOD), and stroke of undetermined cause (SUD) including multiple causes, no identified cause, or incomplete investigation.^[8]^ Subtypes were defined in accordance with risk factors, clinical features, and diagnostic tests. Clinical tests included skull CT, MRI, magnetic resonance angiography, transcranial Doppler, carotid duplex, electrocardiography, and echocardiography. Patients were classified once all required investigations for TOAST had been completed by 2 neurologists who were not involved in the treatment procedure.

### 2.3 National Institute of Health Stroke Scale

The National Institute of Health Stroke Scale (NIHSS) consists of 11 items, each of which grades a related ability with a score of 0 to 4. A score of 0 suggests the ability of this function is normal, whereas other scores indicate some degree of impairment. The individual scores for each item are added together to calculate the total NIHSS score. The maximum total score is 42, which indicates a severe stroke, and the minimum score is 0 (no stroke symptoms). Patients were evaluated upon admission to the hospital by 2 neurologists who were not involved in the treatment procedure.

### 2.4 Data collection

In face-to-face interviews with patients, we collected a variety of demographic data, including age, sex, and history of hypertension, diabetes, coronary vascular disease (CVD), atrial fibrillation (AF), transient ischemic attack, stroke, cerebral hemorrhage, peripheral vascular disease, hypercholesterolemia, and hypertriglyceridemia, along with history of smoking and alcohol consumption. This information was cross-referenced with primary care records. Medical records were used for patients who died during follow-up. Clinical data included blood pressure, blood glucose, serum triglyceride, total cholesterol, high-density lipoprotein cholesterol, low-density lipoprotein (LDL) cholesterol, apolipoprotein, blood urea nitrogen (BUN), uric acid, creatinine, carotid stenosis (>50%), intracerebral hemorrhage, and brain edema.

### 2.5 Follow-up

All patients were followed up by a neurologist at 3 months after the initial admission. The endpoint was defined as any cause of death, with the exception of accidental death.

### 2.6 Statistical analysis

Statistical analysis of the data was carried out using SPSS 20.0 software (IBM, Armonk, NY). For continuous variables, data are presented as mean ± standard deviation, and data for categorical variables are presented as frequencies and percentages. We used the Student *t* test, Chi-square test, and Fisher test to evaluate differences in continuous variables and categorical variables. The associations between potential risk factors and 90-day mortality for each subtype in the TOAST criteria were analyzed with a multiple logistic regression model. We used 95% confidence intervals (CIs) to determine statistical significance. Differences were considered significant when *P* < .05.

## 3 Results

### 3.1 Baseline characteristics and potential risk factors associated with 90-day mortality in stroke patients

Between January 1, 2006 and January 1, 2016, we identified 538 patients with clinical signs attributable to stroke. After comprehensive evaluation, 530 patients were included in the present study; 8 (1.49%) patients were lost because they had registered an incorrect telephone number.

There were 38 deaths during the 90-day follow-up period. The mean age of the surviving population was 68.78 ± 11.22 years, whereas that of the nonsurviving patients was 76.42 ± 7.692 years. Age was found to be a significant risk factor for 90-day mortality following stroke (*P* < .001). In total, 273 (55.49%) patients in the surviving population had a history of hypertension, whereas only 14 (36.84%) patients in the nonsurviving population had hypertension, suggesting that the presence of hypertension was a protective factor against mortality after stroke (*P* = .026). AF occurred in 15 patients in the surviving group (3.05%) and in 5 patients in the nonsurviving population (13.16%). Thus, AF was found to be a significant risk factor for 90-day mortality after stroke (*P* = .002). Other risk factors included high cholesterol (*P* = .003), high BUN (*P* < .001), and high creatinine (*P* = .020) levels. The mean NIHSS score differed significantly between the surviving and nonsurviving populations (5.93 ± 5.74 and 14.82 ± 8.06, respectively; *P* < .001). A greater NIHSS score corresponded to a greater mortality rate, and thus, the NIHSS score was found to be a strong predictor of prognosis after stroke. Significant differences in 90-day mortality were also observed among the different TOAST subtypes (*P* < .001). The highest 90-day mortality among the subtypes was 28.57% for SAO, followed by 12.5% and 10.21% for SOD and LAA, respectively. The lowest mortality rate for stroke was observed in CE patients (0.51%; Table 1).

**Table 1:**
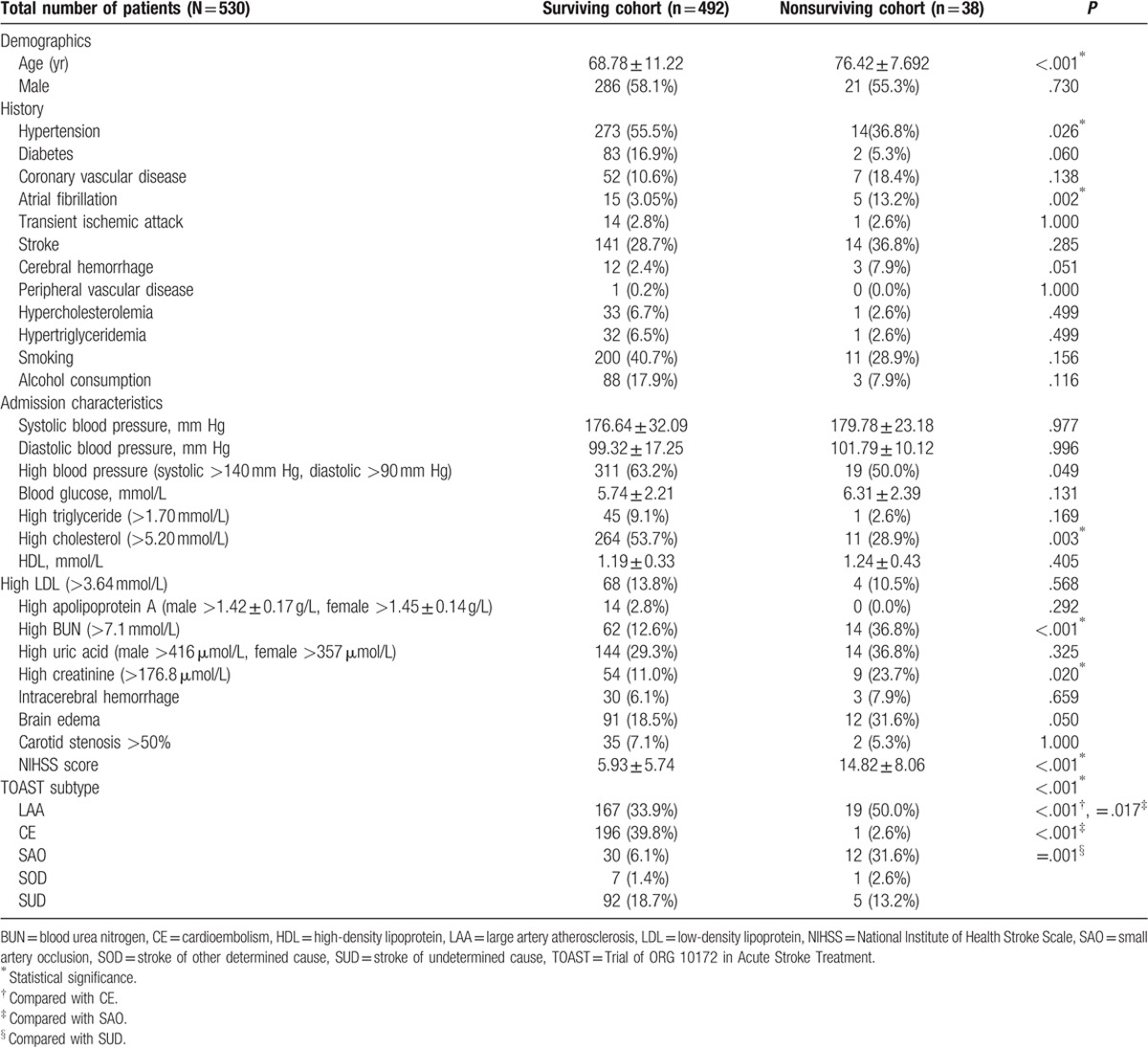
Baseline characteristics and potential risk factors associated with 90-day mortality after stroke.

### 3.2 Multivariate analysis of potential risk factors for 90-day mortality after stroke

Multivariate logistic regression analysis of discrepant factors (Table 2) showed that age (95% CI, 1.031–1.129, *P* = .001), history of hypertension (95% CI, 1.045–5.354, *P* = .039), cholesterol (95% CI, 0.118–0.656, *P* = .003), intracerebral hemorrhage (95% CI, 0.013–0.656, *P* = .003), NIHSS score (95% CI, 1.083–1.209, *P* = .000), and subtypes of SOD and SUN (95% CI = 1.289–15.871, *P* = .018) were associated the increased risk of death within 90 days after stroke.

**Table 2:**
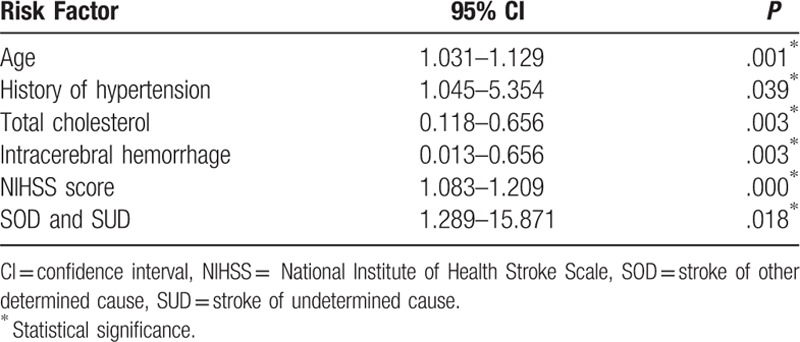
Multivariate analysis of potential risk factors for 90-day mortality after stroke.

### 3.3 Univariate analysis of potential risk factors for 90-day mortality after stroke according to different stroke subtypes classified by TOAST criteria

Univariate analysis was carried out to investigate associations between 90-day mortality and risk factors in different stroke subtypes defined by the TOAST criteria (Table 3). Only 1 case of death occurred in the CE subtype group, and thus preventing univariate analysis of risk factors in this particular subtype. Because of the small number of patients with the SOD subtype (n = 8), we combined the data for SOD and SUD cases. Our analysis revealed different profiles for different subtypes.

**Table 3:**
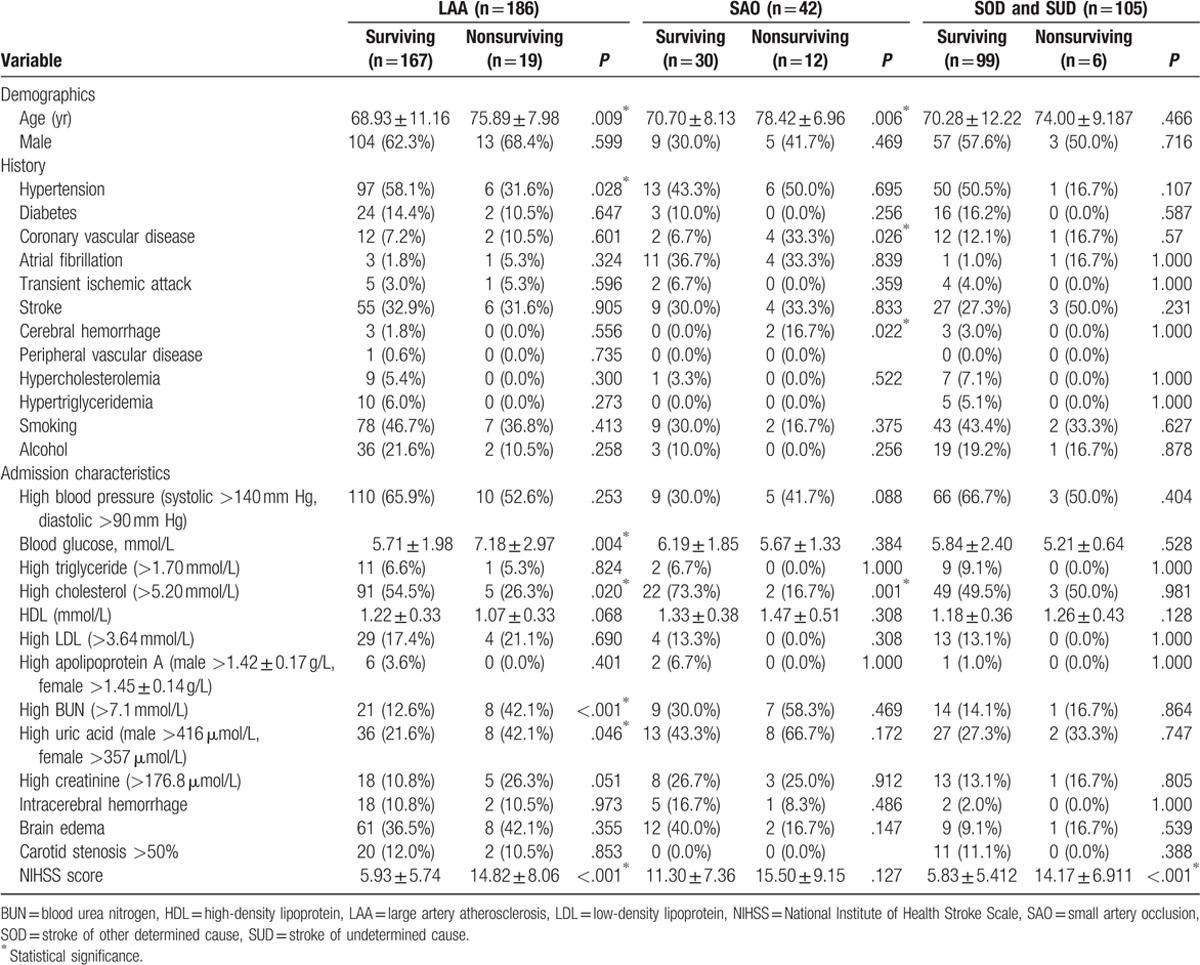
Univariate analysis of the associations between potential risk factors and the 90-day mortality for different stroke subtypes as classified by the Trial of ORG 10172 in Acute Stroke Treatment criteria.

For the LAA subtype, the mean age of the surviving population was 68.93 ± 11.16 years, whereas that of the nonsurviving population was 75.89 ± 7.98 years (*P* = .009). A history of hypertension was present more frequently in the surviving population than in the nonsurviving population among LAA cases (58.08%, *P* = .028). The level of blood glucose in the nonsurviving population of LAA patients was 7.18 ± 2.97 mmol/L, which was higher than that in the surviving population (5.71 ± 1.98 mmol/L; *P* = .004). High cholesterol (*P* = .020), high BUN (*P* < .001), and high uric acid (*P* = .046) levels were more frequent in the nonsurviving population. The mean NIHSS score in the nonsurviving population (14.82 ± 8.06) was greater than that of the surviving population (5.93 ± 5.74; *P* < .001).

Univariate analysis showed that a history of hypertension was associated with SAO. In the SAO subtype, the mean patient age was 78.42 ± 6.96 years in the nonsurviving population and 70.70 ± 8.13 years in the surviving population (*P* = .009). A history of CVD (*P* = .026), history of cerebral hemorrhage (*P* = .022), and a high cholesterol level (*P* = .001) were observed more frequently in the nonsurviving population of SAO cases.

Our analysis also showed that a high NIHSS score was a potential risk factor for 90-day mortality in the SOD and SUD subtypes. The mean NIHSS score in the nonsurviving population was 14.17 ± 6.911, whereas that in the surviving population was 5.83 ± 5.412 (*P* < .001).

### 3.4 Multivariate analysis of potential risk factors for 90-day mortality after stroke according to different stroke subtypes classified by TOAST criteria

Finally, further multivariate analysis of the potential risk factors identified by univariate analysis found independent associations between the 90-day mortality after LAA stroke and age (95% CI, 1.010–1.192, *P* = .028), history of hypertension (95% CI, 3.030–99.136, *P* = .001), blood glucose (95% CI, 1.273–2.354, *P* = .000), cholesterol (95% CI, 0.017–0.462, *P* = .004), uric acid (95% CI, 2.360–64.389, *P* = .003), and NIHSS score (95% CI, 1.076–1.312, *P* = .001). Age (95% CI, 1.012–1.358, *P* = .034) and cholesterol (95% CI, 0.011–0.496, *P* = .007) were also independently associated with 90-day mortality in the SAO subtype of stroke (Table 4).

**Table 4:**
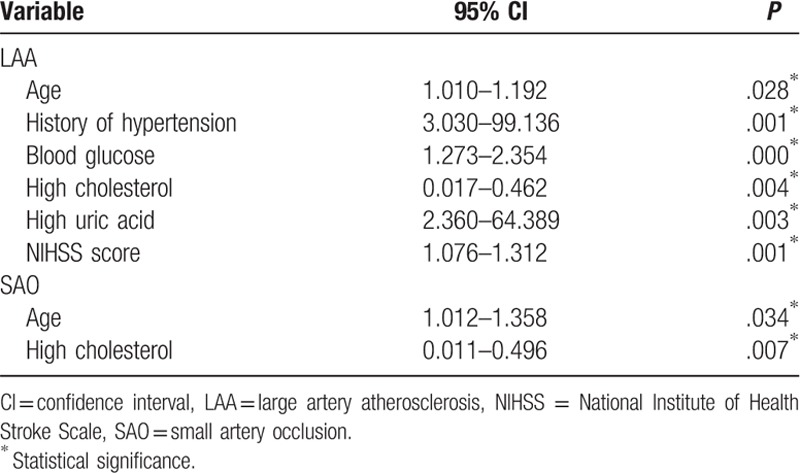
Multivariate analysis of the associations between potential risk factors and the 90-day mortality for different stroke subtypes as classified by the Trial of ORG 10172 in Acute Stroke Treatment criteria.

## 4 Discussion

This retrospective study analyzed 90-day mortality data for different subtypes of ischemic stroke as classified by the TOAST criteria and investigated the potential risk factors that may be involved. Knowledge of how different risk factors relate to mortality among the different TOAST subtypes will be very useful in terms of medical practitioners’ selection of an appropriate course of clinical management, and thus help to reduce mortality.

Our analysis revealed significant differences in 90-day mortality rates among the different TOAST subtypes. Mortality was highest for the SAO subtype (28.57%). The second and third highest mortality rates were observed for the SOD and LAA subtypes (12.5% and 10.21%, respectively). Age was a significant predictor of 90-day mortality in stroke patients. Compared with young patients, the incidences of hypertension and AF were high in elderly patients, and their prognosis was poor.^[9]^ With advanced aging, the structure and function of blood vessels change, and thus impairing the function of vascular endothelial cells. Vascular endothelial cell dysfunction has been shown to be a useful marker of abnormal vascular structure and function, physiological changes that play a crucial role in the incidence and development of stroke.^[10]^

For patients with the LAA subtype, we found that hypertension was a protective factor against mortality, which was higher in patients without hypertension than in those with this condition. Although hypertension was identified as a risk factor in terms of the incidence of stroke, studies have reported there is no relationship between hypertension and death following stroke.^[11]^ The autoregulative ability of large arteries is clearly impaired following stroke, and the recovery of the penumbra relies on an increase in the systolic pressure.^[12]^ Reports have revealed severe stenosis in the large artery of patients with stroke whose resilience was poor. Hypertension was also found to be beneficial in that it increased the blood supply to the penumbra, and thus reducing the size of the infarction and improving prognosis. In addition, Potter et al^[13]^ found that the 90-day mortality of stroke patients in whom hypertension had been controlled following stroke was higher than that of patients who were not treated for hypertension.

In patients who experienced the LAA subtype, the blood glucose level upon admission was significantly higher in the nonsurviving population than in the surviving population. Piironen et al^[14]^ found that hyperglycemia upon admission was relevant to a poor clinical outcome and increased mortality in patients with different types of stroke. Patients with diabetes are susceptible to vessel injury, which is known to be the main cause of the LAA stroke subtype. The reduced resilience of blood vessels negatively affects their contractive function, which can reduce the level of perfusion to the ischemic penumbra.^[15]^ Stenosis and blockage of the cerebral capillaries in patients with diabetes can adversely affect the establishment of collateral circulation, and thus aggravating clinical symptoms.^[16]^ Long-term hyperglycemia can also impair vascular endothelial cells and increase adhesion of the formed elements of the blood, which can cause dysfunction of the circulation and thus exacerbate ischemia. In addition, long-term hyperglycemia can speed the aging process in erythrocytes, causing them to accumulate in the area of infarction, which can ultimately promote the development of stroke. Glucose is able enter neurons and change the osmotic pressure, and thus resulting in osmotic edema and increasing the incidence of irreversible injury to the neurons.^[17]^ Guidelines published by the American Heart Association/American Stroke Association in 2013 for the early management of patients with acute ischemic stroke highlighted that a sustained increase in blood glucose in patients with acute ischemic stroke within 24 hours of onset is indicative of a poor prognosis.^[18]^

A high cholesterol level was observed more frequently in the nonsurviving population of both the LAA and SAO subtypes compared with the surviving populations, suggesting that high cholesterol may be a good predictor of prognosis after stroke. Tirschwell et al^[19]^ also reported that the risk of ischemic stroke was higher in patients with a high total cholesterol quintile than in those with a low quintile (odds ratio [OR] = 1.6, 95% CI, 1.3–2.0), and furthermore, high total cholesterol was strongly associated with atherosclerotic stroke (OR = 3.2) and lacunar stroke (OR = 2.4). High levels of cholesterol in blood vessels increase blood viscosity and aggravate cerebral ischemia and hypoxia, which can adversely affect recovery from stroke. It is also possible that cholesterol could promote the information of the atherosclerosis via oxidative modification, and thus representing the pathophysiological basis of ischemic stroke.^[20]^

High uric acid levels were observed more frequently in the nonsurviving population with the LAA subtype than in the surviving population, which was consistent with the results of a previous report by Bos et al,^[21]^ who reported a strong association between high levels of serum uric acid and the risk of myocardial infarction and stroke. Collectively, these observations suggest that uric acid represents a good predictor for the incidence of myocardial infarction and stroke. It is known that uric acid stimulates the intima of vessels and cause local inflammation, which is involved in the formation and development of atherosclerosis.^[22]^ Furthermore, serum uric acid is a type of oxidant that can promote the production of LDL cholesterol, which could then activate the formation of atherosclerosis.^[23]^ In addition, uric acid could potentially block the self-repair of vascular endothelial cells by inhibiting the differentiation of vascular endothelial stem cells.^[24]^

NIHSS is known to be useful for both clinical prognosis and investigative research of stroke.^[25]^ Studies have shown that patients with a baseline NIHSS score >16 are more likely to die, whereas those with a score <6 are more likely to recover from stroke.^[26]^ In this study, the mean NIHSS score in the nonsurviving population was higher than that in the surviving population, suggesting that the NIHSS score is a significant predictor of the prognosis of stroke. Another study previously reported a strong association between the NIHSS score and both functional outcome and mortality in acute stroke patients treated with tissue-type plasminogen activator.^[27]^

Finally, 1 limitation of our study was selection bias in our patient population. This study included only patients attending our hospital. We were not able to include patients with minor stroke who did not attend our hospital or patients who died before the start of the study. Information regarding patients’ history was obtained from the interview and medical records, which might have introduced recall bias. Moreover, previous studies have found that some important polymorphisms such as PlA2,^[28,29]^ CaMK4,^[30]^ and G-protein-coupled receptor kinase^[31]^ are linked to hypertension and stroke. Because technical limitations related to the detection technique, we did not include such an analysis in our current study, which might create another form of bias.

## Acknowledgments

The authors have no acknowledgements to declare.

## Author contributions

**Conceptualization:** Weimin Wei, Suting Li.

**Data curation:** Weimin Wei, Suting Li, Fulan San, Shijun Zhang, Jianjun Guo, Li Zhang.

**Formal analysis:** Weimin Wei, Suting Li, Fulan San, Shijun Zhang.

**Investigation:** Weimin Wei, Fulan San, Qingyu Shen, Jianjun Guo.

**Methodology:** Weimin Wei, Suting Li, Fulan San, Shijun Zhang.

**Project administration:** Weimin Wei, Jianjun Guo, Li Zhang.

**Resources:** Weimin Wei, Suting Li, Shijun Zhang, Jianjun Guo, Li Zhang.

**Software:** Weimin Wei, Suting Li, Fulan San, Shijun Zhang.

**Supervision:** Weimin Wei, Suting Li, Fulan San, Qingyu Shen, Jianjun Guo, Li Zhang.

**Validation:** Weimin Wei, Suting Li, Fulan San, Qingyu Shen, Jianjun Guo.

**Visualization:** Weimin Wei, Suting Li, Fulan San, Jianjun Guo.

**Writing – original draft:** Weimin Wei, Suting Li.

**Writing – review & editing:** Weimin Wei, Jianjun Guo, Li Zhang.
